# Impact of endogenous LH and LH supplementation on clinical outcome following a long GnRH agonist protocol in younger and older patients

**DOI:** 10.3389/fendo.2026.1635317

**Published:** 2026-03-30

**Authors:** Jing Chen, Huiliu Fan, Qiuyue Wen, Yuan Ou, Xiaoxia Wu, Pinxiu Huang, Qiuyan Huang, Dingyuan Zeng

**Affiliations:** 1 Center of Reproductive Medicine, Guangzhou Women and Children’s Medical Center-Liuzhou Hospital, Liuzhou, Guangxi, China; 2 Guangxi Clinical Research Center for Obstetrics and Gynecology, Liuzhou, Guangxi, China; 3 Center of Reproductive Medicine, Liuzhou Maternity and Child Healthcare Hospital, Guangxi, China; 4 Affiliated Maternity Hospital and Affiliated Children’s Hospital of Guangxi University of Science and Technology, Liuzhou, Guangxi, China; 5 Center of Reproductive Medicine, The First Affiliated Hospital of Guilin Medical University, Guilin, Guangxi, China; 6 Center of Reproductive Medicine, Affiliated Hospital of Youjiang Medical University for Nationalities, Baise, Guangxi, China; 7 Key Laboratory of Clinical Diagnosis and Treatment Research of High-Incidence Diseases in Guangxi, Baise, Guangxi, China

**Keywords:** clinical outcome, controlled ovarian stimulation, hCG triggerday, long protocol, luteinizing hormone

## Abstract

**Background:**

The optimal luteinizing hormone (LH) concentration on the day of hCG trigger during long-protocol IVF/ICSI remains uncertain. Although some studies associate profound LH suppression with impaired reproductive outcomes, others report no adverse effects. Importantly, most existing evidence does not consider maternal age as a potential effect modifier, despite its well established role in ovarian response and oocyte competence.

**Methods:**

This retrospective cohort study included 9,979 IVF/ICSI cycles performed between 2009 and 2017, comprising 8,786 cycles in women younger than 38 years and 1,193 cycles in women aged 38 years or older. First, cycles were stratified into five groups according to serum LH concentration on the day of hCG trigger: <0.5, 0.5 to <1.0, 1.0 to <2.0, 2.0 to <5.0, and ≥5.0 IU/L, to evaluate associations with clinical outcomes. Second, among cycles with LH ≤1.0 IU/L on stimulation day 7, when r-hLH supplementation was considered according to institutional protocol, we compared outcomes between those who received r-hLH and those who did not, adjusting for age, antral follicle count, BMI and basal FSH. The primary outcomes were good-quality embryo rate, clinical pregnancy rate, and live birth rate.

**Results:**

In young patients (<38 years), LH levels on the hCG trigger day were not associated with oocyte yield, embryo quality, clinical pregnancy, or live birth rates, and r-hLH supplementation conferred no benefit. In older patients (≥38 years), LH <0.5 IU/L was associated with a lower good-quality embryo rate and greater gonadotropin consumption with prolonged stimulation, reflecting deeper pituitary suppression; however, it was not linked to differences in live birth rates, and r-hLH supplementation was not associated with a statistically significant improvement in good-quality embryo rate after multivariable adjustment.

**Conclusions:**

In women <38 years, profound LH suppression does not impair IVF outcomes and r-hLH is unnecessary. In those ≥38 years, low LH correlates with poorer embryo quality, but r-hLH supplementation shows no significant benefit after adjustment. Routine r-hLH add-back based solely on low LH levels is not supported.

## 1 Introduction

Since the early 1990s, the application of assisted reproductive technology (ART) has progressively improved clinical pregnancy rates among infertile patients, largely due to advances in controlled ovarian stimulation (COS) protocols (1). The introduction of gonadotropin-releasing hormone agonist (GnRH-a) represents a milestone in COS, and its long-protocol regimen, initiated in the luteal phase, has become widely adopted in China for *in vitro* fertilization (IVF) and intracytoplasmic sperm injection (ICSI). This approach initially induces a flare-up effect before achieving pituitary desensitization, thereby preventing premature luteinizing hormone (LH) surges, enhancing follicular synchronization, increasing oocyte yield, and improving embryo quality (2).

However, despite its widespread use, the optimal serum LH concentration during controlled ovarian hyperstimulation (COH) remains controversial. LH is indispensable for appropriate steroidogenesis and dominant follicle selection, as outlined by the classic two-cell theory: theca cells require LH to produce androgens, which serve as substrates for granulosa cell aromatization into estradiol under FSH stimulation (3). Numerous studies have demonstrated that both endogenous and exogenous FSH are critical for follicular recruitment, selection, and growth, from preantral stages through to dominance (4–7). In fact, in women with hypogonadotropic hypogonadism (HH), FSH alone can drive follicular development, but this is consistently associated with suboptimal estradiol production, underscoring the non-redundant role of LH in complete folliculogenesis (3).

The use of GnRH-a suppresses endogenous LH to non-physiological levels, raising concerns about impaired oocyte and embryo quality. The “LH threshold and ceiling” theory posits that follicular maturation requires LH concentrations above a minimal threshold to activate paracrine signaling between theca and granulosa cells, yet below an upper limit beyond which excessive androgen exposure may be detrimental (8, 9). However, empirical evidence remains conflicting: some studies report that low LH on the hCG trigger day adversely affects oocyte competence and pregnancy outcomes (10), while others find no association (11) or even suggest better outcomes with lower LH (12). These discrepancies may stem from heterogeneous patient populations, variable LH assay methodologies, and, critically, the failure to account for age-related differences in ovarian reserve and compensatory capacity.

Importantly, serum LH concentration may not accurately reflect its *in vivo* bioactivity. In HH patients, for example, circulating LH often remains below 1.0 IU/L even after exogenous supplementation, yet follicular responses vary widely, highlighting the influence of LH isoform composition, receptor sensitivity, and intraovarian feedback mechanisms (13). Given that ovarian aging alters both gonadotropin dynamics and follicular responsiveness, it is essential to stratify analyses by age when evaluating the impact of LH suppression.

Therefore, this study aims to investigate the association between serum LH levels on the hCG trigger day and IVF/ICSI outcomes in long-protocol cycles, with explicit stratification by age (<38 versus ≥38 years). By integrating clinical, embryological, and live birth data, we seek to clarify whether low LH represents a modifiable deficiency or merely a biomarker of diminished ovarian reserve, and ultimately inform evidence-based decisions regarding r-hLH supplementation.

## 2 Materials and methods

### 2.1 Patients

This retrospective study included patients who underwent IVF/ICSI procedures at the Centre of Reproductive Medicine at Liuzhou Maternity and Child Healthcare Hospital in Guangxi Province, China, between 2009 and 2017. All participants provided informed consent after receiving counseling for infertility treatments and routine IVF/ICSI procedures.

The exclusion criteria were as follows: (i) endometrial abnormalities, such as endometrial polyps, intrauterine adhesions, uterine submucosal myomas, or chronic endometritis; (ii) adenomyosis; (iii) hydropic fallopian tubes; (iv) polycystic ovarian syndrome; or (v) stage III or higher endometriosis.

This study retrospectively analyzed a total of 9,979 IVF/ICSI cycles performed between 2009 and 2017, which included 8,786 cycles from young patients (<38 years old) and 1,193 cycles from older patients (≥38 years old). Within each age group, cycles were further classified into five subgroups according to the serum LH levels on the hCG trigger day, as follows: LH < 0.5 IU/L, 0.5 ≤ LH < 1.0 IU/L, 1.0 ≤ LH < 2.0 IU/L, 2.0 ≤ LH < 5.0 IU/L, and LH ≥ 5.0 IU/L. This stratification allowed for an assessment of the impact of different LH levels on IVF/ICSI outcomes across distinct age groups.

**Figure 1:**
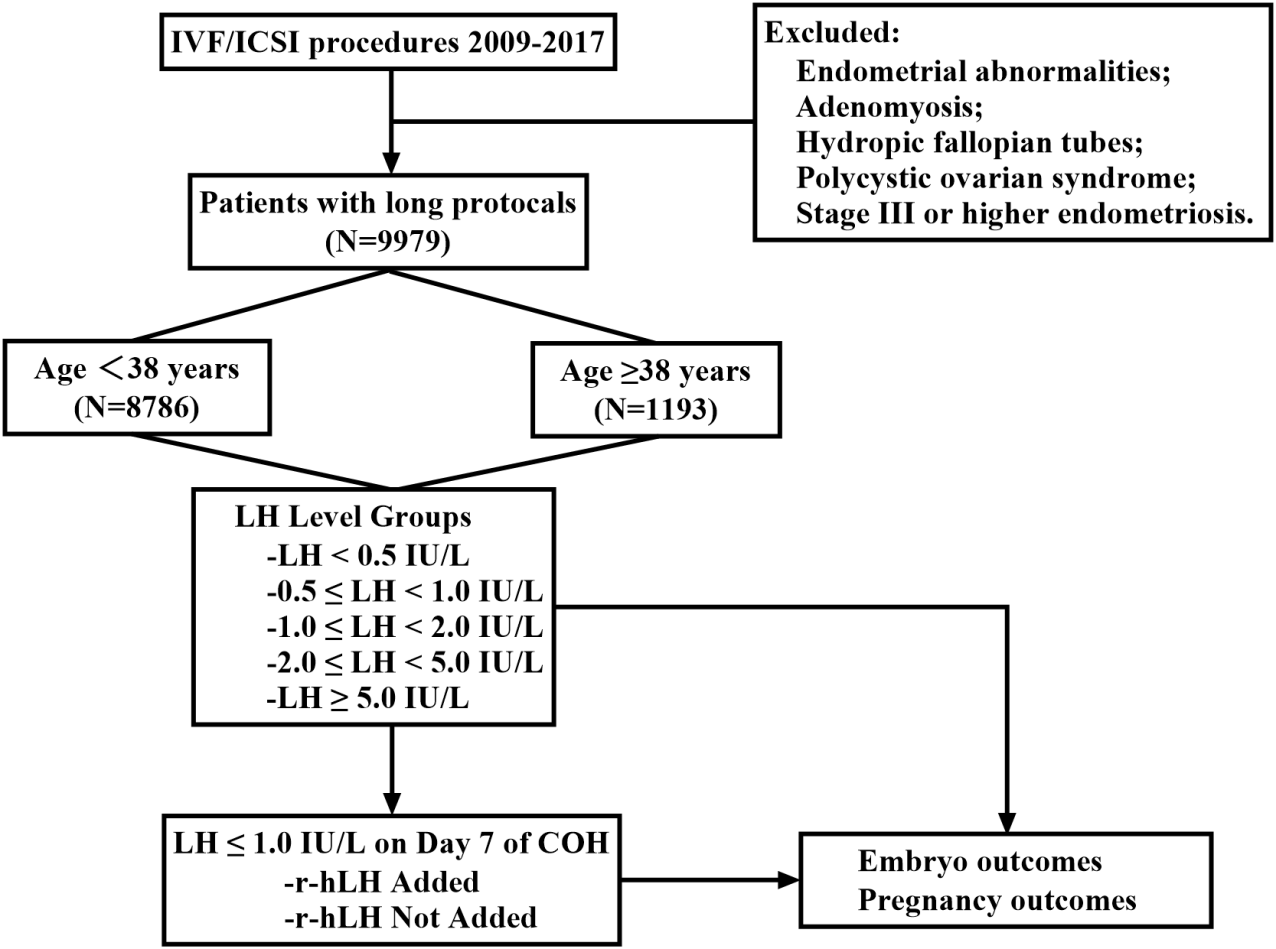
Flowchart of patient inclusion and stratification in the study. A total of 9,979 IVF/ICSI cycles performed between 2009 and 2017 using a long GnRH agonist protocol were initially identified. Cycles were excluded due to endometrial abnormalities, adenomyosis, hydrosalpinx, polycystic ovary syndrome (PCOS), or stage III–IV endometriosis. The remaining 9,979 cycles were included in the final analysis and stratified by maternal age into two groups: <38 years (n = 8,786) and ≥38 years (n = 1,193). These cohorts were further analyzed according to luteinizing hormone (LH) levels on the day of hCG trigger and r-hLH supplementation status.

In addition, the young (*n* = 4022 cycles) and old patients (*n* = 268 cycles) whose LH level was ≤1.0 on the 7th day of controlled ovarian hyperstimulation (COH) were divided into two groups, respectively, depending on whether LH was added or not: in the young patients—Recombinant hLH added(r-hLH, Luveris, Merck Group, Germany; 75 IU daily until the hCG trigger day) and r-hLH not added; in the old patients—LH added and LH not added. **Figure 1** illustrates the entire study process.

### 2.2 IVF/ICSI treatment

Patients in the GnRH-a protocol group underwent long downregulation, initiated in the midluteal phase, using continuous-release triptorelin acetate 1.875 mg i.m. (Triptorelin Acetate for Injection; Ipsen, France). The criteria for successful downregulation were as follows: follicles < 0.5 cm in diameter, endometrial thickness < 5.0 mm, serum FSH < 5.0 IU/L, LH < 5.0 IU/L, and serum estradiol (E2) < 50.0 pg/ml. Following pituitary suppression, daily administration of recombinant FSH (Gonal-f, Merck Group, Germany) was initiated and subsequently adjusted according to follicle size and hormonal changes. Oocyte maturation was triggered with 5000–10,000 U hCG (Lizhu, Zhuhai, China) when at least three follicles reached 18.0 mm in diameter. Oocytes were retrieved 36 hours after hCG administration, and IVF/ICSI was performed as indicated. Routine embryo transfer was performed under ultrasound guidance 3 days after oocyte retrieval. Luteal phase support was initiated with progesterone the night following oocyte retrieval. Pregnancies were detected 12 days after embryo transfer by serum hCG measurement (biochemical pregnancy) and confirmed as clinical pregnancy by ultrasound detection of embryonic heart beat. Luteal support was continued until the 10th week of pregnancy.

Embryo quality was assessed at 72 hours (3rd day) after insemination or ICSI, based on morphology scores that considered the number and symmetry of blastomeres and the percentage of extracellular fragmentation, using a modified version of the scoring system described by recent studies. The good-quality embryo rate was calculated as the number of good-quality embryos divided by the number of fertilized oocytes. Clinical pregnancy was confirmed by the presence of an intrauterine gestational sac and fetal heart rate on ultrasound examination. The spontaneous abortion rate was defined as pregnancy loss occurring before 12 weeks of gestation.

### 2.3 Observation

The following parameters were analyzed for all patients: age, infertility duration, body mass index (BMI), number of baseline antral follicles, basal FSH levels, duration and dose of gonadotropin (Gn) used, serum LH levels, E2 levels on the day of hCG injection, number of oocytes retrieved, endometrial thickness on the day of oocyte retrieval, high-quality embryo rates, clinical pregnancy rates, and live birth rates. Serum LH concentrations were measured using a chemiluminescent microparticle immunoassay (CMIA) on the Abbott Architect i2000 system (Abbott Laboratories, Chicago, IL, USA). The LH assay has a functional sensitivity of 0.09 IU/L and a reportable range of 0.12–250.00 IU/L. The total imprecision (coefficient of variation) is ≤7% for LH levels ≤70 IU/L and ≤10% for levels >70 IU/L; notably, within the clinically relevant range of 0.5–1.0 IU/L, the assay imprecision is ≤0.07 SD.

### 2.4 Statistical analysis

Statistical analysis was performed using SPSS (v.24.0; IBM SPSS Statistics, IBM Corporation, Armonk, NY, USA), and the data were presented as mean ± standard deviation or number (%). Group comparisons across the five LH strata (<0.5, 0.5 to <1.0, 1.0 to <2.0, 2.0 to <5.0, and ≥5.0 IU/L) were initially assessed by one-way ANOVA or chi-square test. To evaluate the association between LH level on hCG trigger day and live birth rate within each age group (<38 and ≥38 years), multivariable logistic regression models were fitted, adjusting for antral follicle count, BMI, basal FSH, and duration of infertility. Separately, among cycles with LH ≤1.0 IU/L on stimulation day 7, the effect of r-hLH supplementation on good-quality embryo rate was analyzed using analysis of covariance (ANCOVA), with the same covariates included in the model. A p-value < 0.05 was considered statistically significant.

## 3 Results

Baseline characteristics—including age, BMI, basal FSH, and antral follicle count (AFC)—were generally comparable across the five LH subclasses on the hCG trigger day in both age groups (**Tables 1**, **2**). Patients with profound LH suppression (LH < 0.5 IU/L) required significantly higher total gonadotropin doses and longer stimulation durations compared to those in all other LH subclasses, in both younger (<38 years) and older (≥38 years) age groups.

**Table 1:** The clinical characteristics among the five groups (different LH level of the trigger day of HCG) of <38 years.

Variables	LH<0.5 (n=1459)	0.5≤LH<1 (n=3056)	1≤LH<2 (n=3144)	2≤LH<5 (n=993)	LH≥5 (n=134)	P-value
Age (years)	31.67 ± 3.45	31.74 ± 3.42	31.79 ± 3.42	31.80 ± 3.40	31.69 ± 3.00	0.521
Duration of infertility (years)	6.79 ± 3.67	7.00 ± 3.67	6.87 ± 3.78	6.85 ± 3.63	6.77 ± 3.90	0.432
BMI (kg/m^2^)	22.14 ± 3.19	22.77 ± 3.12	22.32 ± 2.91	22.97 ± 2.64	22.45 ± 2.64	0.645
No. of antral follicles	12.74 ± 4.64	12.13 ± 4.70	12.03 ± 4.38	12.84 ± 4.02	12.60 ± 4.59	0.987
Basal FSH (IU/l)	6.99 ± 1.70	6.83 ± 2.11	7.08 ± 1.92	7.01 ± 2.11	7.13 ± 1.88	0.065
Total Gn dose^a^	31.90 ± 10.84*	29.38 ± 10.29	28.29 ± 9.84	27.94 ± 9.75	25.19 ± 7.52	0.003
Duration of Gn (days)	12.51 ± 1.82*	11.59 ± 1.70	11.00 ± 1.50	10.60 ± 1.48	10.56 ± 1.62	0.018
Endometrial thickness (mm)	12.77 ± 2.81	12.66 ± 2.88	12.63 ± 2.63	12.74 ± 2.40	12.56 ± 2.41	0.765
LH Post-Regulation(IU/l)	0.43 ± 0.28	0.62 ± 0.36	0.84 ± 0.58	1.25 ± 1.00	1.69 ± 1.16	0.006
LH of hCG trigger(IU/l)	0.31 ± 0.13	0.75 ± 0.14	1.37 ± 0.27	2.80 ± 0.73	8.31 ± 4.09	0.008
E2 of hCG trigger(pg/ml)	3259.46 ± 1584.74	3254.60 ± 1654.97	3193.23 ± 1538.35	3276.44 ± 1527.71	3352.99 ± 204933.49	0.076
No. of oocytes retrieved	12.68 ± 5.33	12.29 ± 5.16	12.00 ± 4.81	12.54 ± 4.74	12.76 ± 4.85	0.089
No. of embryos transferred	1.68 ± 0.51	1.70 ± 0.53	1.75 ± 0.54	1.80 ± 0.50	1.78 ± 0.46	0.436
High-quality embryo rate (%)	48.20% (5926/12300)	48.60% (11790/24280)	47.80% (10842/22692)	47.90% (2937/6247)	49.30% (453/430)	0.095
Clinical pregnancy rates (%)	57.6% (840/1459)	56.3% (1722/3056)	58.1% (1825/3144)	54.8% (544/993)	60% (81/134)	0.599
Abortion rates (%)	12.7% (107/840)	12.9% (222/1722)	13.0% (237/1825)	12.3% (67/544)	9.9% (8/81)	0.937
OHSS rates (%)	2.12% (31/1459)	2.65% (81/3056)	2.86% (90/3144)	1.41% (14/993)	3.73% (5/134)	0.077
Live birth rates(%)	50.2% (732/1459)	48.9% (1495/3056)	50.3% (1580/3144)	47.8% (475/993)	54.5% (73/134)	0.433

*Indicates compared with other groups, p<0.05 a Number of 75IU ampoules.

**Table 2:** The clinical characteristics among the five groups (different LH level of the trigger day of HCG) of ≧38 years.

Variables	LH<0.5 (n=134)	0.5≤LH<1 (n=364)	1≤LH<2 (n=522)	2≤LH<5 (n=154)	LH≥5 (n=19)	P-value
Age (years)	39.41 ± 1.78	39.58 ± 1.65	39.71 ± 1.67	39.84 ± 2.02	39.31 ± 1.20	0.789
Duration of infertility (years)	5.51 ± 5.26	5.74 ± 4.89	5.56 ± 5.04	5.60 ± 4.91	5.68 ± 3.68	0.765
BMI(kg/m^2^)	22.44 ± 2.83	22.66 ± 3.13	22.16 ± 2.58	22.08 ± 5.31	22.50 ± 2.11	0.654
No. of antral follicles	10.16 ± 3.92	10.32 ± 3.70	10.56 ± 3.30	10.18 ± 2.81	10.21 ± 2.20	0.787
Basal FSH (IU/l)	6.94 ± 1.78	6.87 ± 1.77	6.95 ± 1.99	6.96 ± 2.61	6.30 ± 2.16	0.675
Total Gn dose^a^	39.29 ± 11.63*	36.46 ± 10.99	33.87 ± 8.96	31.00 ± 9.38	28.88 ± 7.34	0.002
Duration of Gn (days)	12.33 ± 2.11*	11.43 ± 1.68	10.88 ± 1.40	10.10 ± 1.38	9.64 ± 1.37	0.003
Endometrial thickness (mm)	12.04 ± 2.68	12.26 ± 2.62	12.03 ± 2.62	12.09 ± 2.72	12.55 ± 1.88	0.734
LH Post-Regulation(IU/l)	0.54 ± 0.26	0.65 ± 0.36	0.77 ± 0.74	0.98 ± 0.87	1.28 ± 1.59	0.004
LH on trigger day (IU/l)	0.32 ± 0.13	0.77 ± 0.14	1.38 ± 0.26	2.74 ± 0.72	9.84 ± 5.18	0.006
E2 on hCG Day (pg/ml)	2727.36 ± 1625.40	2817.01 ± 1459.79	2677.69 ± 1573.85	2693.64 ± 1406.52	2662.02 ± 1015.86	0.457
No. of oocytes retrieved	11.08 ± 5.35	10.62 ± 4.90	10.90 ± 4.59	10.86 ± 3.67	10.58 ± 4.10	0.089
No. of embryos transferred	1.91 ± 0.61	1.84 ± 0.58	1.81 ± 0.57	1.84 ± 0.57	1.79 ± 0.63	0.096
High-quality embryo rate (%)	43.3%* (418/964)	46.40% (1155/2487)	45.40% (1277/2812)	46.10% (304/659)	48.80% 59/121	0.012
Clinical pregnancy rates (%)	37.3% (50/134)	41.2% (150/364)	36.4% (190/522)	31.8% (49/154)	52.6% (10/19)	0.173
Abortion rates (%)	26.0% (13/50)	30.7% (46/150)	24.7% (47/190)	30.6% (15/49)	50% (5/10)	0.381
OHSS rates (%)	1.50% (2/134)	1.10% (4/364)	0.57% (3/522)	0 (0/154)	0 (0/19)	0.543
Live birth rates(%)	27.6% (37/134)	28.6% (104/364)	27.4% (143/522)	22.1% (34/154)	26.3% (5/19)	0.66

*Indicates compared with other groups, *p* < 0.05.

a Number of 75IU ampoules.

**Figure 2:**
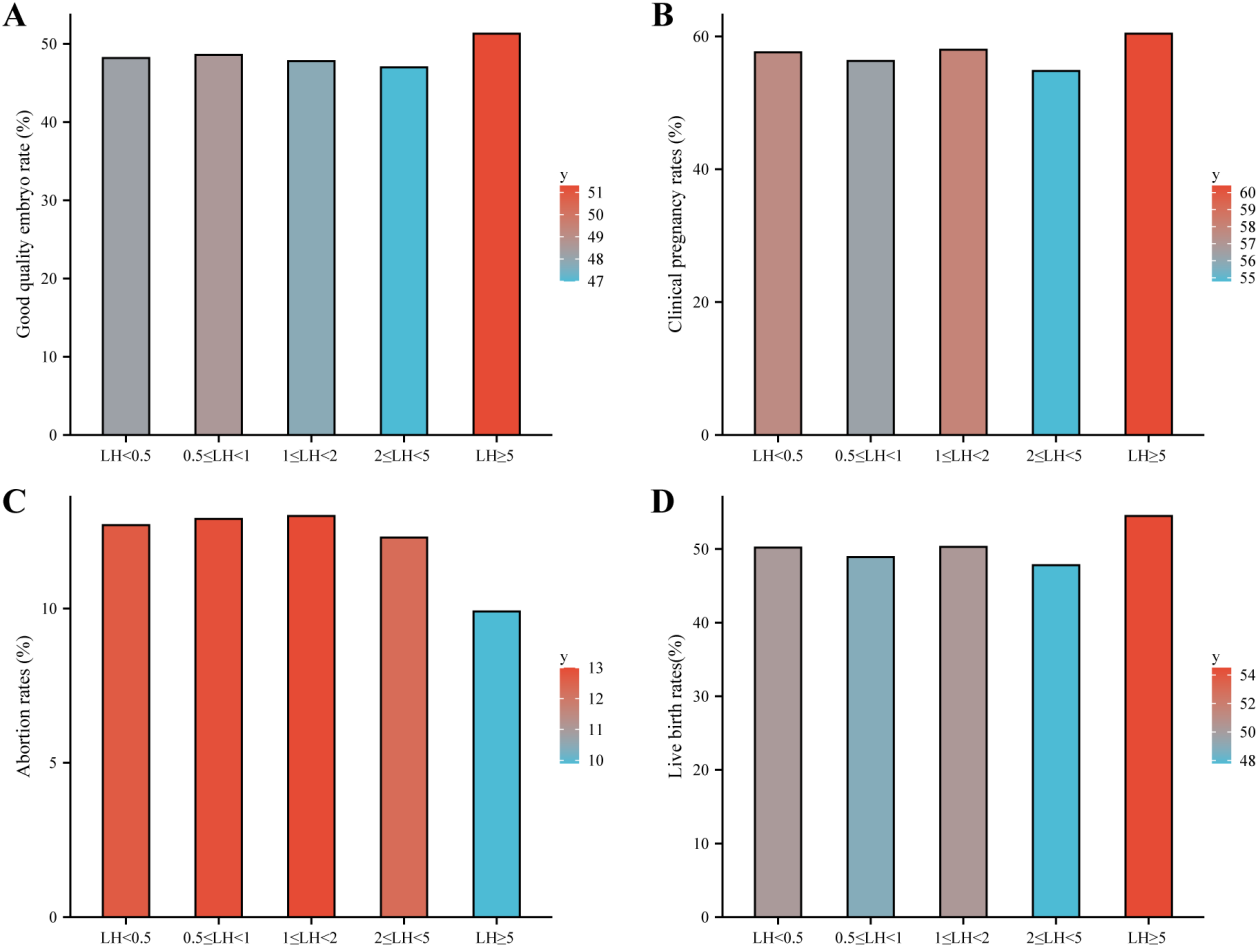
Clinical outcomes across LH subclasses on the hCG trigger day in women aged <38 years. Bar plots show **(A)** good-quality embryo rate, **(B)** clinical pregnancy rate, **(C)** miscarriage rate, and **(D)** live birth rate among five LH subclasses: LH<0.5, 0.5≤LH<1.0, 1.0≤LH<2.0, 2.0≤LH<5.0, and LH≥5.0 IU/L. No significant differences were observed for any outcome across groups.

Among women <38 years, clinical outcomes—including number of oocytes retrieved, high-quality embryo rates, clinical pregnancy rates, abortion rates, and live birth rates—did not differ significantly across LH subclasses (**Table 1**). The main clinical outcomes are visualized in **Figure 2**. Consistently, multivariable logistic regression showed no independent association between LH subclass and live birth after adjusting for age, AFC, BMI, basal FSH, and infertility duration (overall p = 0.394; **Table 3**). Compared to the reference group (LH < 0.5 IU/L), adjusted odds ratios (aORs) for other LH subclasses ranged from 0.81 to 0.87 (all p > 0.20). Age and AFC remained strong positive predictors of live birth (aOR per year: 0.95, 95% CI 0.93–0.96; aOR per AFC unit: 1.02, 95% CI 1.01–1.03; both p < 0.001).

**Table 3:** Adjusted association between LH subclass on the hCG trigger day and live birth among women aged <38 years.

Variable	Adjusted OR (95% CI)	P-value
LH subclass on hCG trigger day		
*(Reference: LH < 0.5 IU/L)*		
0.5 ≤ LH < 1.0 IU/L	0.81 (0.62–1.05)	0.245
1.0 ≤ LH < 2.0 IU/L	0.80 (0.61–1.04)	0.2
2.0 ≤ LH < 5.0 IU/L	0.87 (0.64–1.17)	0.414
LH ≥ 5.0 IU/L	0.81 (0.56–1.16)	0.252
Age (per year increase)	0.95 (0.93–0.96)	<0.001
Antral follicle count (AFC, per unit)	1.02 (1.01–1.03)	<0.001
BMI (kg/m²)	1.00 (0.98–1.02)	0.904
Basal FSH (IU/L)	0.99 (0.97–1.01)	0.448
Duration of infertility (years)	0.99 (0.97–1.01)	0.116

OR, odds ratio; CI, confidence interval; LH, luteinizing hormone; BMI, body mass index; FSH, follicle-stimulating hormone.

Model adjusted for all listed covariates simultaneously.

**Figure 3:**
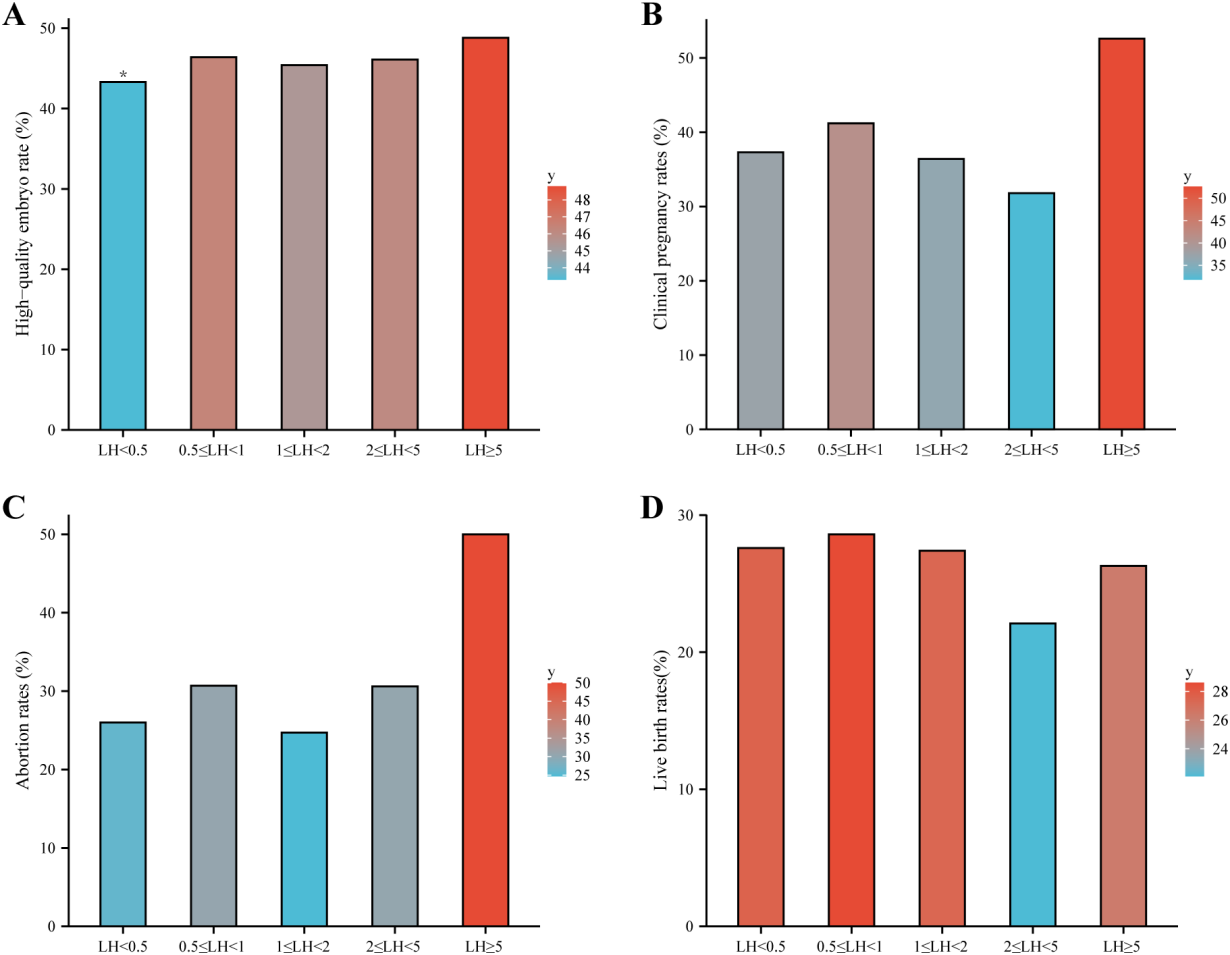
Clinical outcomes across LH subclasses on the hCG trigger day in women aged ≥38 years. Bar plots show **(A)** good-quality embryo rate, **(B)** clinical pregnancy rate, **(C)** miscarriage rate, and **(D)** live birth rate among five LH subclasses: LH<0.5, 0.5≤LH<1.0, 1.0≤LH<2.0, 2.0≤LH<5.0, and LH≥5.0 IU/L. The good-quality embryo rate was significantly lower in the LH <0.5 IU/L group (p < 0.05), while other outcomes did not differ significantly across groups.

In women ≥38 years, the LH < 0.5 IU/L group had a significantly lower high-quality embryo rate than other subclasses, while clinical pregnancy, miscarriage, and live birth rates were similar across groups (**Table 2**). The main clinical outcomes are visualized in **Figure 3**. Multivariable analysis confirmed that LH subclass was not independently associated with live birth (overall p = 0.788; **Table 4**). Age was the dominant predictor of live birth (aOR per year: 0.76, 95% CI 0.73–0.79, p < 0.001).

**Table 4:** Adjusted association between LH subclass on the hCG trigger day and live birth among women aged ≥38 years.

Variable	Adjusted OR (95% CI)	P-value
LH subclass on hCG trigger day		
*(Reference: LH < 0.5 IU/L)*		
0.5 ≤ LH < 1.0 IU/L	1.07 (0.64–1.80)	0.901
1.0 ≤ LH < 2.0 IU/L	1.20 (0.72–1.99)	0.736
2.0 ≤ LH < 5.0 IU/L	1.22 (0.74–2.01)	0.709
LH ≥ 5.0 IU/L	0.94 (0.53–1.67)	0.908
Age (per year increase)	0.76 (0.73–0.79)	<0.001
Antral follicle count (AFC, per unit)	1.00 (0.99–1.01)	0.899
BMI (kg/m²)	1.05 (0.89–1.09)	0.071
Basal FSH (IU/L)	0.96 (0.92–1.00)	0.231
Duration of infertility (years)	0.98 (0.96–1.00)	0.091

OR, odds ratio; CI, confidence interval; LH, luteinizing hormone; BMI, body mass index; FSH, follicle-stimulating hormone.

Model adjusted for all listed covariates simultaneously.

In both age groups, r-hLH supplementation (restricted to patients with LH ≤1.0 IU/L on day 7) did not increase LH levels beyond 1.0 IU/L on the hCG trigger day and had no impact on E_2_ dynamics or stimulation duration (**Tables 5**, **6**). Among women <38 years, r-hLH supplementation did not improve any clinical outcome when baseline LH was ≤1.0 IU/L on stimulation day 7 (**Table 5**). However, it was associated with a higher high-quality embryo rate and a non-significant increase in live birth among older women (**Table 6**). However, the good-quality embryo rate did not differ significantly between those who received r-hLH supplementation and those who did not after adjustment for age, antral follicle count (AFC), body mass index (BMI), basal follicle-stimulating hormone (FSH), and duration of infertility (adjusted mean difference: 7.5%, 95% CI: −2.3% to 17.3%, P = 0.136). Notably, advancing maternal age was independently associated with a lower good-quality embryo rate (β = −2.38 per year, P = 0.035), whereas other covariates showed no statistically significant effects (**Table 7**).

**Table 5:** the clinical characteristics among the two groups (add R-hLH or not) of <38 years.

Variables	Add R-hLH (n=3915)	not R-hLH (n=107)	P-value
Age (years)	30.38 ± 3.12	30.43 ± 4.01	0.569
Duration of infertility (years)	4.50 ± 3.04	4.35 ± 3.00	0.634
BMI (kg/m^2^)	21.98 ± 4.41	21.82 ± 3.10	0.456
No. of antral follicles	12.56 ± 4.75	12.53 ± 4.48	0.546
Basal FSH (IU/l)	6.74 ± 1.97	6.44 ± 1.75	0.874
Total Gn dose^a^	29.26 ± 10.12	28.90 ± 5.78	0.653
Duration of Gn (days)	11.90 ± 1.80	11.55 ± 1.28	0.778
Endometrial thickness (mm)	12.63 ± 3.07	12.46 ± 2.43	0.668
d7-LH (IU/l)^b^	0.65 ± 0.86	0.70 ± 0.38	0.076
LH on trigger day (IU/l)	0.61 ± 0.25	0.71 ± 0.20	0.084
E2 on hCG Day (pg/ml)	3219.30 ± 1576.65	3240.97 ± 1607.94	0.267
No. of oocytes retrieved	12.59 ± 5.15	12.30 ± 5.38	0.367
No. of embryos transferred	1.66 ± 0.51	1.78 ± 0.54	0.095
High-quality embryo rate (%)	48.48% (15921/32841)	48.65% (468/964)	0.34
Clinical pregnancy rates (%)	58.26% (2281/3915)	58.88% (63/107)	0.899
Abortion rates (%)	12.45% (284/2281)	11.11% (7/63)	0.765
OHSS rates (%)	2.61% (102/3915)	7.48% (8/107)	0.002
Live birth rates (%)	50.63% (1982/3915)	51.40% (55/107)	0.874

^a^Number of 75IU ampoules.

^b^Serum levels on the 7th day of controlled ovarian hyperstimulation.

**Table 6:** The clinical characteristics among the two groups (add R-hLH or not) of ≧38 years.

Variables	Add R-hLH (n=210)	not R-hLH (n=58)	P-value
Age (years)	39.64 ± 1.62	39.97 ± 1.77	0.674
Duration of infertility (years)	6.42 ± 4.80	6.26 ± 5.97	0.187
BMI (kg/m^2^)	22.14 ± 3.31	22.14 ± 2.48	0.549
No. of antral follicles	10.09 ± 3.71	9.88 ± 3.21	0.458
Basal FSH (IU/l)	6.74 ± 1.61	6.50 ± 1.46	0.178
Total Gn dose^a^	36.08 ± 10.63	35.35 ± 10.49	0.086
Duration of Gn (days)	11.64 ± 1.88	11.21 ± 1.86	0.375
Endometrial thickness (mm)	12.29 ± 2.74	12.28 ± 2.38	0.542
d7-LH (IU/l)^b^	0.66 ± 0.45	0.76 ± 0.46	0.096
LH on trigger day (IU/l)	0.75 ± 0.18	0.62 ± 0.27	0.167
E2 on hCG Day (pg/ml)	2771.77 ± 1455.05	2763.90 ± 1837.47	0.834
No. of oocytes retrieved	10.94 ± 5.30	10.52 ± 4.76	0.678
No. of embryos transferred	1.61 ± 0.52	1.81 ± 0.63	0.056
High-quality embryo rate (%)	47.89% (703/1468) ^*^	40.42% (154/381)	0.009
Clinical pregnancy rates (%)	40.00% (84/210)	34.48% (20/58)	0.445
Abortion rates (%)	27.38% (23/84)	30.00% (6/20)	0.814
OHSS rates (%)	0.47% (1/210)	1.72% (1/58)	0.328
Live birth rates (%)	29.05% (61/210)	24.14% (14/58)	0.461

*Indicates compared with other groups, *p* < 0.05.

^a^Number of 75IU ampoules.

^b^Serum levels on the 7th day of controlled ovarian hyperstimulation.

**Table 7:** Adjusted association between r-hLH supplementation and good-quality embryo rate in women aged ≥38 years: results from analysis of covariance (ANCOVA).

Variable	Adjusted Mean difference (β)	Standard error	*t*	P-value
r-hLH supplementation (yes vs. no)	7.50	5.01	1.5	0.136
Age (per 1-year increase)	-0.38	1.11	-2.14	0.035
Antral follicle count (per 1 unit)	0.80	0.75	1.06	0.29
BMI (per 1 kg/m^2^ increase)	-0.12	0.55	-0.22	0.828
Basal FSH (per 1 IU/L increase)	-0.94	0.76	-1.22	0.226
Duration of infertility (per 1 year)	-0.26	0.63	-0.42	0.677

Dependent variable: good-quality embryo rate (%).

All models were adjusted for all covariates listed above.

Total sample size = 268 cycles (Group 1: n = 58; Group 2: n = 210). Model R² = 0.109; adjusted R² = 0.062.

r-hLH, recombinant human luteinizing hormone; BMI, body mass index; FSH, follicle-stimulating hormone.

## 4 Discussion

In women younger than 38 years, profound LH suppression, even below 0.5 IU/L, did not impair oocyte yield, embryo quality, or live birth rates, and r-hLH supplementation conferred no benefit when initiated at LH ≤1.0 IU/L on stimulation day 7. These findings underscore the resilience of ovarian function in this age group and support the adequacy of standard long-protocol GnRH agonist regimens, even under deep pituitary desensitization. Notably, both total gonadotropin consumption and stimulation duration increased significantly as LH levels declined, reflecting a state of relative ovarian resistance rather than functional failure. This adaptive response aligns with Westergaard’s observation that the degree of pituitary suppression induced by GnRH agonists depends on the formulation, dosage, and route of administration (4). Crucially, follicular development remains largely preserved despite minimal LH, a phenomenon explained by the “spare receptor hypothesis” proposed by Chappel and Howles (5). According to this model, even trace amounts of circulating LH are sufficient to occupy enough luteinizing hormone receptors (LHR) on theca cells to maintain baseline androgen production required for aromatization and folliculogenesis. Moreover, FSH may compensate for low LH by enhancing paracrine signaling within the follicle and upregulating LHR expression in granulosa cells, thereby amplifying sensitivity to low systemic LH concentrations.

In contrast, women aged 38 years or older exhibited heightened sensitivity to profound LH suppression. Those with LH levels <0.5 IU/L on the hCG trigger day demonstrated a significantly lower good-quality embryo rate compared to those with higher LH concentrations, suggesting that age-related declines in ovarian reserve may impair the follicle’s capacity to compensate for minimal gonadotropin signaling. This observation is consistent with Wei et al., who reported reduced clinical pregnancy rates in long-protocol cycles when LH fell below 1.12 IU/L on the trigger day (14). However, this embryological disadvantage did not translate into measurable differences in clinical pregnancy, miscarriage, or live birth rates across LH strata—a finding supported by other studies showing no predictive value of post-downregulation LH for pregnancy outcomes (15, 16), and even reports that LH levels below 0.5 IU/L do not adversely affect follicular development or pregnancy rates in long protocols (17, 18).

When r-hLH supplementation was initiated in cycles with LH ≤1.0 IU/L on stimulation day 7 to rescue follicular steroidogenesis, the unadjusted good-quality embryo rate was higher. However, after multivariable adjustment for age, antral follicle count, BMI, basal FSH, and duration of infertility, this difference was no longer statistically significant. This attenuation implies that low LH primarily reflects diminished ovarian responsiveness rather than a directly correctable deficiency. Indeed, while some meta-analyses suggest that r-hLH co-treatment improves implantation and clinical pregnancy rates in women aged 35–40 years (1), this benefit appears to vanish after age 40 (9, 13). The loss of efficacy likely stems from a fundamental shift in the primary barrier to live birth: beyond advanced reproductive age, embryonic aneuploidy rather than gonadotropin insufficiency becomes the dominant determinant of reproductive failure. Consequently, even if exogenous LH modestly enhances follicular androgenesis or oocyte cytoplasmic maturation, it cannot overcome the high burden of chromosomal abnormalities, explaining why improvements in intermediate embryological markers rarely culminate in higher live birth rates.

The diminished responsiveness to gonadotropins in older women likely stems from multiple, interrelated alterations in the hypothalamic–pituitary–ovarian axis that compromise follicular competence even before ovulation. First, advancing age is associated with a shift in circulating LH toward less bioactive glycoforms characterized by reduced sialylation and sulfonation, which exhibit lower receptor-binding affinity and diminished steroidogenic potency despite normal immunoreactive concentrations (6). Second, the intricate paracrine crosstalk between theca and granulosa cells, which relies on androgen precursors and local growth factors such as IGF-1 and AMH, progressively deteriorates with ovarian aging (7). This erosion of intraovarian signaling diminishes the follicle’s ability to buffer systemic LH deficiency through local compensatory mechanisms. Third, in the context of diminished ovarian reserve, granulosa cells often display downregulated expression of luteinizing hormone/choriogonadotropin receptors (LHCGR), a change linked to elevated basal FSH and blunted estradiol production (3). LHCGR expression inversely correlates with serum LH levels in older women, suggesting a maladaptive feedback loop that further desensitizes the follicle to available LH (3). Together, these age-dependent changes impair theca cell androgen synthesis, depriving aromatase in granulosa cells of its essential substrate and ultimately disrupting both follicular development and oocyte cytoplasmic maturation. The resulting suboptimal follicular microenvironment may explain why exogenous r-hLH, although capable of modestly elevating systemic LH, fails to fully restore embryological quality or reproductive outcomes in this population.

In light of these findings, clinical strategies for pituitary suppression should be tailored to ovarian age rather than serum LH levels alone. Women younger than 38 years exhibit robust intraovarian compensatory mechanisms that maintain folliculogenesis even under profound LH suppression; consequently, routine reduction of the standard 1.875 mg triptorelin dose solely to preserve LH is unnecessary. In contrast, for women aged ≥38 years—particularly those with diminished ovarian reserve—a more cautious approach may be warranted. A modest reduction in GnRH agonist dose (e.g., to 1.0–1.25 mg) could be considered as a precautionary measure to avoid imposing additional stress on an already compromised follicular environment, though its impact on live birth remains unproven. Ultimately, the intensity of pituitary suppression should be guided by dynamic markers of follicular response (e.g., follicle growth trajectory and estradiol rise), not isolated LH concentrations.

This study has several limitations. Its retrospective, single-center design precluded randomization and may be susceptible to residual confounding, despite multivariable adjustment for key covariates including age, antral follicle count, BMI, basal FSH, and duration of infertility. Data were derived from a single academic center that used standardized protocols, such as a fixed GnRH agonist dose and a uniform policy of fresh embryo transfer, which may limit generalizability to settings with more heterogeneous clinical practices. However, this consistency in ovarian stimulation and luteal-phase support enhanced internal validity by minimizing treatment-related variability, thereby strengthening confidence in the observed associations between LH dynamics and embryological outcomes. Additionally, our analysis was restricted to live birth rates from fresh embryo transfers; cumulative live birth data incorporating frozen-thawed cycles could not be reliably reconstructed due to transitions in electronic medical record systems during the 2009–2017 study period. Finally, serum LH concentration, while clinically practical, does not reflect isoform-specific bioactivity, a factor that may partially account for the limited efficacy of r-hLH supplementation in older women.

In summary, in women younger than 38 years, profound LH suppression during long-protocol IVF/ICSI does not impair outcomes, and r-hLH supplementation is unnecessary. In women aged 38 years or older, although LH levels below 0.5 IU/L are associated with lower good-quality embryo rates, r-hLH supplementation initiated at LH ≤1.0 IU/L on stimulation day 7 did not significantly improve embryo quality or live birth rates after adjustment for key covariates. Thus, routine r-hLH add-back based solely on low serum LH levels is not supported by current evidence.

## Data Availability

The raw data supporting the conclusions of this article will be made available by the authors, without undue reservation.
